# Skin-Reducing Mastectomy and Direct-to-Implant Breast Reconstruction Without Mesh: A Single-Institute Experience

**DOI:** 10.7759/cureus.81417

**Published:** 2025-03-29

**Authors:** Ahmed E Abdelmageed, Ahmed T Awad, Tarek A El-Fayoumi, Mahmoud A Alhussini, Mahmoud O Shalaby, Ahmed A Abdelkader

**Affiliations:** 1 Department of General Surgery, Portsmouth Hospitals University NHS Trust, Portsmouth, GBR; 2 Surgical Oncology Unit, Faculty of Medicine, Alexandria University, Alexandria, EGY; 3 Department of General Surgery, Faculty of Medicine, Arab Academy for Science, Technology and Maritime Transport, Alexandria, EGY

**Keywords:** breast cancer, dermal sling, implant reconstruction, macromastia, skin reducing mastectomy

## Abstract

Introduction: Performing breast reconstruction in large and ptotic breasts remains technically challenging, with a high incidence of postoperative complications and unsatisfactory cosmetic results. Skin-reducing mastectomy (SRM) was introduced for breast reconstruction for females with macromastia. In this study, we report the experience in Alexandria Main University Hospital (AMUH), Alexandria, Egypt, with SRM and direct-to-implant (DTI) breast reconstruction without the utilization of biological or synthetic meshes.

Patients and methods: A prospective study was carried out at AMUH from October 2020 to October 2022. It included 20 female patients having large and ptotic breasts who were indicated for mastectomy, with the exclusion of non-motivated patients, inflammatory breast cancer, significant clinical comorbidities, and patients indicated for postoperative radiotherapy. All the operations were done as a single stage in which the SRM was performed through a Wise-pattern skin incision, along with the creation of a dermal sling to cover the implant.

Results: A total of 20 surgeries were performed, in which the implant was placed in a pre-pectoral position in 10 cases. In the other 10 cases, the implant was inserted partially subpectoral by creating a musculo-dermal pouch to envelop the implant. The most prevalent complication encountered was delayed wound healing at the area of the inverted T-junction. None of the studied patients experienced wound infection, capsular contracture, or implant loss.

Conclusion: SRM and DTI breast reconstruction is a feasible technique that can be safely used in patients with large and ptotic breasts. The inferior dermal sling allows for a single-stage implant-based breast reconstruction without the utilization of biological or synthetic meshes.

## Introduction

Breast cancer is the most prevalent cancer in women worldwide. In 2020, nearly 2.3 million new breast cancer cases were diagnosed worldwide. Lifetime risk of developing breast cancer is 12.4% or one in eight women [1,2]. With advancements in breast cancer diagnosis and treatment, along with improved survival rates, mastectomies followed by breast reconstruction have become increasingly common [3]. Reconstruction can be performed either immediately or at a later stage using autologous tissue flaps or prosthetic implants [4].

In 1991, skin-sparing mastectomy (SSM) was introduced by Toth and Lappert as an attempt for preservation of the skin envelop to facilitate immediate breast reconstruction [5]. SSM was classified by Carlson in 1997 according to the type and site of incision into four types; types I, II, and III can be used for small and medium-sized breasts and are carried out through peri-areolar incisions [6]. Performing SSM in large ptotic breasts has been technically challenging with a high incidence of suboptimal cosmetic results and post-operative complications, which may be related to inadequate blood supply for such large skin envelopes [7]. Therefore, Type IV SSM, known as skin-reducing mastectomy (SRM), was introduced for large and ptotic breasts. It is carried out via an inverted T or Wise-pattern skin incisions to allow for reduction of the skin envelope [8].

Implant-based breast reconstruction remains the most frequently used reconstructive method for breast cancer [9]. The optimal plane for implant placement remains debated, with subpectoral and prepectoral approaches each having advantages and drawbacks. The subpectoral technique offers implant protection and a lower risk of capsular contracture but may lead to more blood loss from muscle detachment, loss of pectoralis major muscle function, longer operative times, and animation deformity. In contrast, the prepectoral approach is less invasive, preserves muscle function, and reduces pain and animation deformity but requires adequate skin flap vascularity to avoid complications such as necrosis and implant exposure. In addition, capsular contracture and skin rippling are more common than in the subpectoral group. Proper patient selection and intraoperative vascular assessment are crucial for successful outcomes [10-12].

Acellular dermal matrix (ADM) and synthetic meshes were introduced to solve many of these problems. They can be used to support the implant in prepectoral or subpectoral positions, reducing capsular contracture and improving cosmetic outcomes. However, their high cost and limited availability prevent widespread use in all institutions [13-15].

This study aims to report the experience of Alexandria Main University Hospital (AMUH) in Alexandria, Egypt, with SRM and direct-to-implant (DTI) breast reconstruction as a surgical treatment option for female patients with operable breast cancer and large, ptotic breasts, without the use of ADM or synthetic meshes.

## Materials and methods

This prospective study was conducted at AMUH between October 2020 and October 2022. The study was approved by the Ethical Committee of the Faculty of Medicine, Alexandria University, on October 15, 2020 (IRB number: 00012098 - FWA number: 00018699). The patients’ records were kept confidential, and all the patients signed informed consent before being enrolled in the study.

The study included 20 female patients having large and ptotic breasts who were indicated for mastectomy. The patients were followed up until April 2024, with a median follow-up of 30 months. Exclusion criteria were non-motivated patients, inflammatory breast cancer, significant clinical comorbidities, and patients indicated for adjuvant radiotherapy.

Surgical procedure

**Figure 1:**
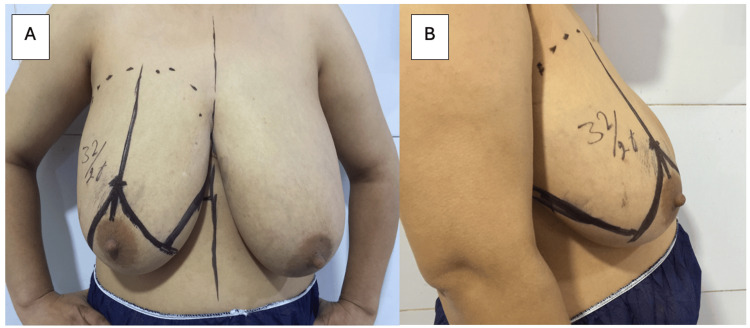
Preoperative marking for skin reducing mastectomy (A: Anterior view, B: Lateral view) The distance from the supra-sternal notch to the nipple is 32 cm. The future nipple is planned within 2 cm below the inframammary fold, which is now positioned 26 cm from the supra-sternal notch. From this point, the Wise-pattern was designed by drawing two oblique lines, each measuring 9 cm, with an angle of 90 degrees between them.

Preoperative marking of the breast was done with the patient in an upright position for defining the breast borders, breast meridian, inframammary fold (IMF), and the site of the future nipple. Typically, the future nipple was positioned up to 2 cm below the IMF. Starting from this future nipple point, the Wise-pattern was designed by drawing two oblique lines measuring 7-9 cm, with a 60-90 degree in between, depending on the amount of skin to be excised. The endpoints of those two oblique lines were then extended laterally and medially to join the IMF (Figure 1).

For all the patients, antibiotics were administered upon anaesthesia induction. SRM was performed by making skin incisions along the marked design. The area enclosed in the Wise-pattern below the nipple-areola complex (NAC) was then de-epithelized to make the dermal sling. After that, the formal mastectomy was carried out by dissection along the subcutaneous plane while preserving the dermal vasculature and second intercostal perforator medially. Sentinel lymph node biopsy (SLNB) was performed through the same incision. Intra-operative frozen section was used to assess the positivity of the SLNB. Patients with positive SLNB were excluded from the study as they underwent reconstruction using a tissue expander for the potential need for adjuvant radiotherapy. Patients with negative SLNB proceeded with implant-based reconstruction.

**Figure 2:**
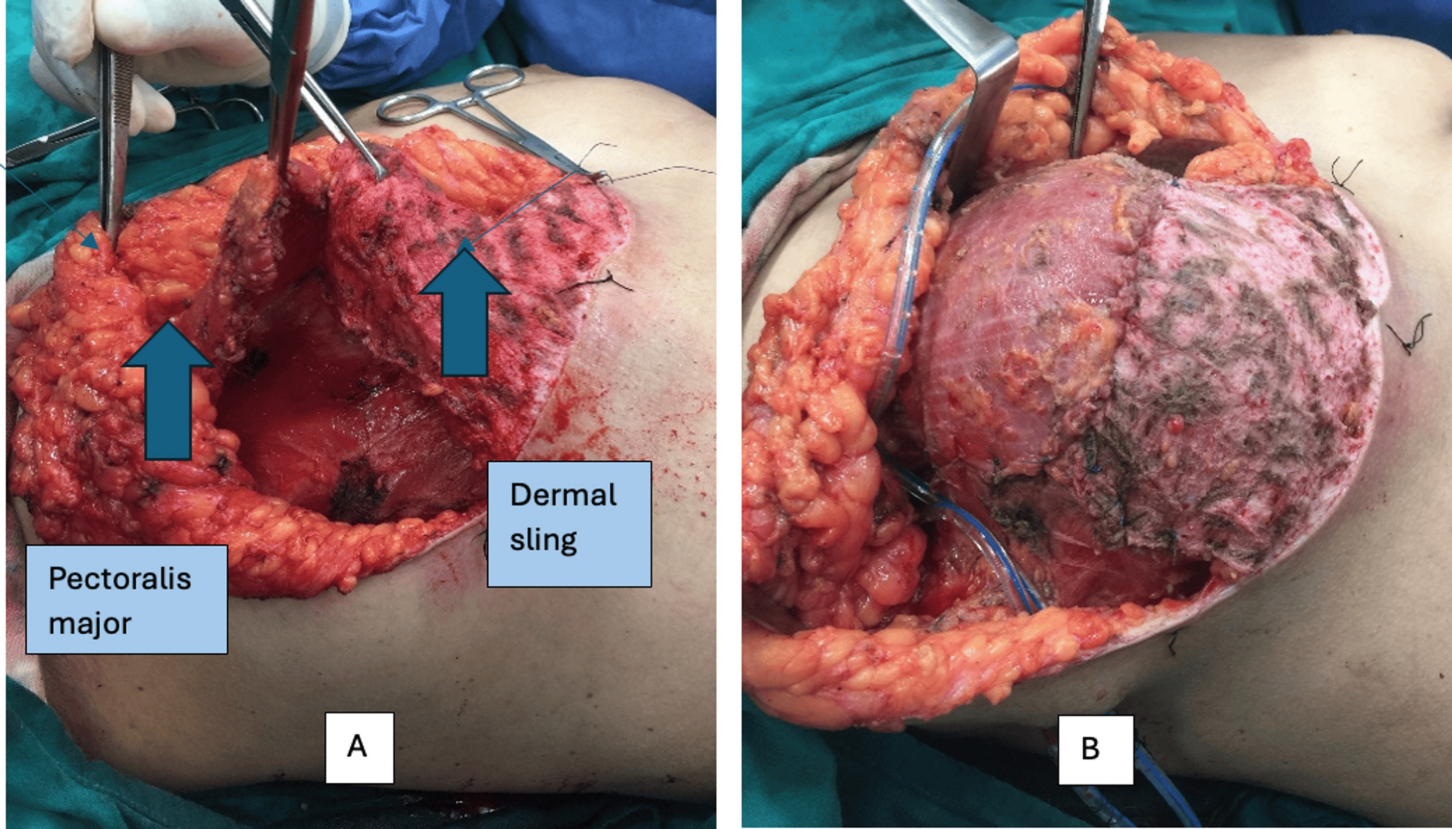
(A) Intra-operative picture showing the dermal sling, the dissected pectoralis major muscle and the pocket; (B) The implant placed in the subpectoral pocket after suturing the dermal sling to the detached lower border of the pectoralis major muscle

**Figure 3:**
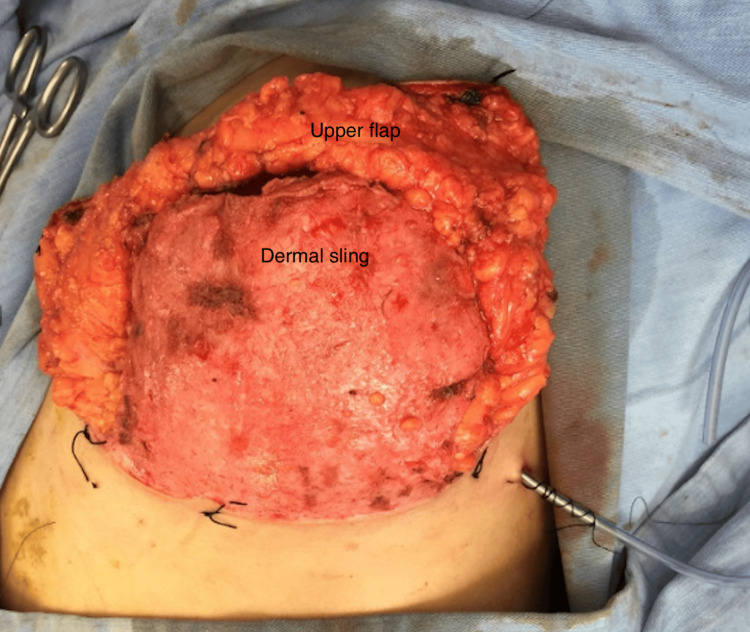
Intra-operative picture showing SRM and pre-pectoral implant insertion (inserted only under the dermal sling) SRM: skin-reducing mastectomy

The decision regarding the detachment of the pectoralis major muscle was made depending on the thickness of the skin flaps and the length of the dermal sling. In patients with thin mastectomy skin flaps, dissection of the pectoralis major was done by detaching its lower border from the chest wall, followed by suturing it to the upper edge of the dermal sling. This creates a dermo-muscular pouch to envelop the implant. In cases where the skin flaps were adequately thick with sufficient length of the dermal sling, the implant was inserted in a pre-pectoral position, solely covered by the dermal sling, which was sutured to the upper flap's dermis (Figures 2, 3). 

Breast sizers were used to check for the pocket size before inserting the implants. Rounded smooth implants were used in all cases. Closed suction drains were inserted beneath the skin flaps and were removed when the amount of seroma was less than 50 ml for two consecutive days. The patients were offered contralateral breast symmetrisation, whether by mastopexy or reduction mammoplasty. All the patients were discharged from the hospital on the next day.

Assessment of specimen and monitoring

A pathological assessment of the excised mastectomy specimen was performed to determine the histopathological type, tumour grade, tumour size, nodal status, lymphovascular invasion, and hormonal receptors. The following data were recorded: operative time, postoperative complications, and cosmetic outcome. The patients were monitored weekly for the first three weeks, then monthly for the next three months, and subsequently every six months. The evaluation of the cosmetic outcome involved assessments by both the surgical team and the patient herself. Standardized postoperative digital photographs were taken in different views for comparison and follow-up. The surgical team included independent reviewers who did not participate in the operation to evaluate the following items: breast shape and contour, formation of the IMF, breast consistency, and the overall result. Patients’ evaluation was done by asking the patient to give a score from one to four according to the degree of satisfaction about the result of the operation.

Data analysis

Data were analyzed using IBM SPSS Statistics for Windows, Version 20.0 (Released 2011; IBM Corp., Armonk, New York, United States). Qualitative data were described using numbers and percentages. Quantitative data were described using range (minimum and maximum), mean, standard deviation, median, and interquartile range (IQR).

## Results

Table 1 illustrates the demographic data of the studied patients, with the mean age being 44.40 ± 10.05 years. All the studied patients had breast cup sizes of D or above. Five patients received neoadjuvant systemic treatment, while 15 patients did not have any neoadjuvant treatment. All the studied patients were non-smokers.

**Table 1:** Patient characteristics (N=20)

Patient characteristics	Values
Age (years), mean ± SD (range)	44.40 ± 10.05 (30-63)
BMI (kg/m^2^), mean ± SD (range)	28.80 ± 2.24 (24.0-32.0)
Medical illness, n	
Negative	12
Hypertension	5
Asthma	2
Autoimmune disease	1
Breast cup size, n	
D	7
DD/E	7
DDD/F	4
G	1
H	1
Degree of ptosis, n	
Moderate (Grade 2)	3
Severe (Grade 3)	17
Neoadjuvant treatment, n	
Yes	5
No	15

The mean distance from the suprasternal notch to the nipple was 29.65 ± 3.80 cm. The minimum distance was 24 cm, and the maximum distance was 43 cm. The implant was inserted in a pre-pectoral position, solely under the dermal sling in 10 cases (50%). In the remaining 10 cases (50%), the implant was inserted in a dermo-muscular pocket by detaching the lower fibres of the pectoralis major and suturing it to the dermal sling (Figures 2, 3). The mean size of the used implants was 477.25 ± 79.48 cc. The size of the smallest implant was 325 cc, while the largest measured 615 cc.

All the patients were offered contralateral breast symmetrizing procedures. Only two patients opted for breast symmetrisation (10%); one had contralateral reduction mammoplasty, and the other underwent contralateral mastopexy. The remaining 18 patients refused contralateral symmetrisation. The mean operative time was 150 ± 29.63 minutes for the surgery without symmetrizing procedure and was 210 ± 22.4 minutes when bilateral procedure was done.

**Figure 4:**
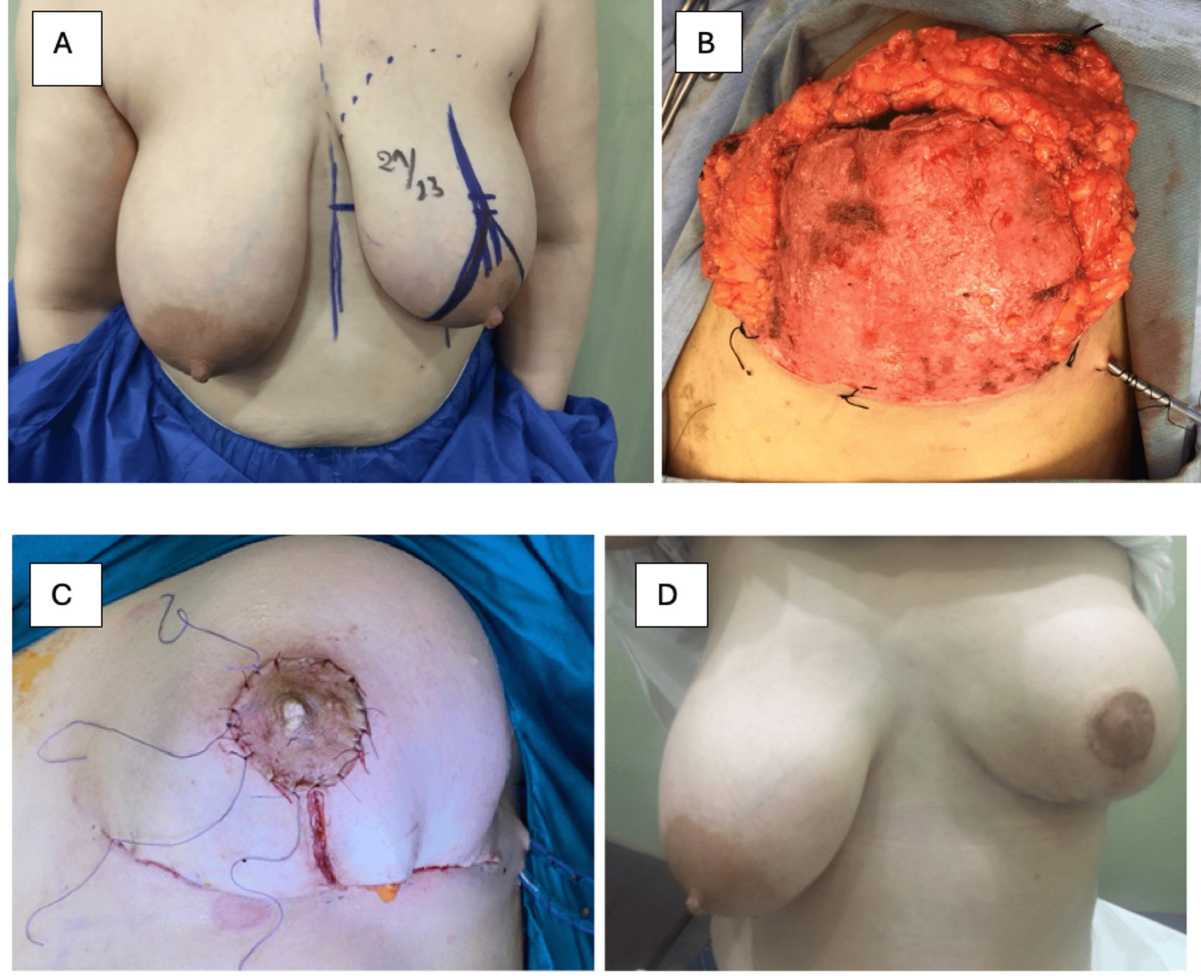
(A) Preoperative marking for SRM; (B) Pre-pectoral implant insertion and covering with the dermal sling; (C) Intra-operative photograph showing the nipple-areola graft; (D) Three month postoperative after SRM and pre-pectoral direct to implant reconstruction and NAC graft SRM: skin-reducing mastectomy; NAC: nipple-areola complex

**Figure 5:**
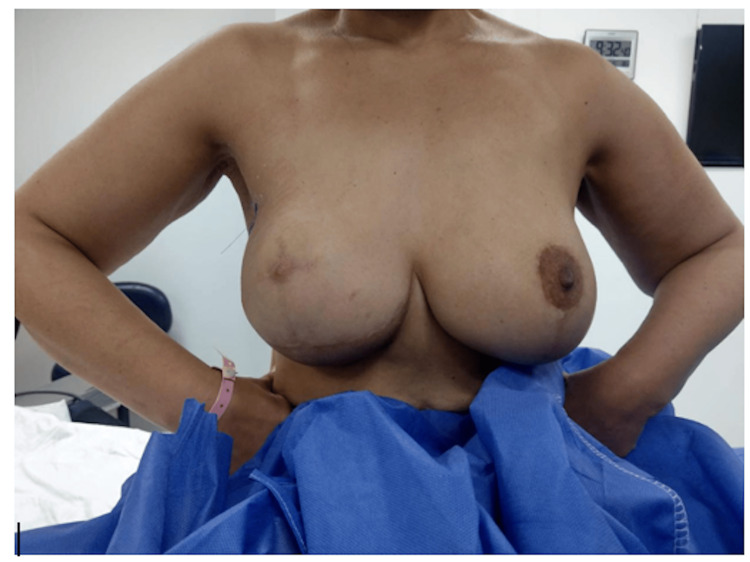
Excellent cosmetic results (one year postoperative for left mastopexy, right SRM, implant reconstruction followed by secondary nipple reconstruction) SRM: skin-reducing mastectomy

NAC preservation and reconstruction were done in 5 cases (20%), three of which had a nipple graft, performed by de-epithelizing the areola at the beginning of the operation, to be grafted at the end of the operation after insertion of the implant and closure of the skin. Remarkably, these three patients exhibited an excellent cosmetic outcome (Figure 4). One patient had a secondary nipple reconstruction in a second session using a local tissue flap from the same breast (Figure 5). In the remaining patient, nipple-areola preservation was attempted based on superior and inferior dermal pedicles. However, one week postoperative, the areola became necrotic, necessitating its removal with primary wound closure.

Regarding postoperative complications, 16 cases (80%) had a smooth postoperative course, and only four cases (20%) encountered complications. The most frequent complication was delayed healing of the wound at the area of the T-junction, encountered in two patients and was conservatively managed by wound dressing. No additional interventions were required beyond standard wound care. Both cases healed well with simple wound dressings, with complete healing achieved within an average of three to four weeks. Nipple necrosis was encountered in one patient who underwent preservation of the areola based on superior and inferior dermal pedicles. This patient needed excision of the nipple-areola with primary wound closure in a second session. One patient experienced implant displacement noticed in the first follow-up visit. Initially, the implant had been placed solely under the dermal sling. Then, it was displaced from its upper boundary and shifted laterally. To manage this, a second surgery was performed where the implant was placed beneath the pectoralis major muscle, which was detached and sutured to the dermal sling. None of the studied cases experienced wound infection, haematoma, capsular contracture, or implant loss (Table 2). 

**Table 2:** Distribution of the studied cases according to complications (N = 20)

Complications	Frequency	Percentage
No	16	80%
Yes	4	20%
Delayed healing of the T-junction	2	10%
Nipple necrosis	1	5%
Implant displacement	1	5%

**Figure 6:**
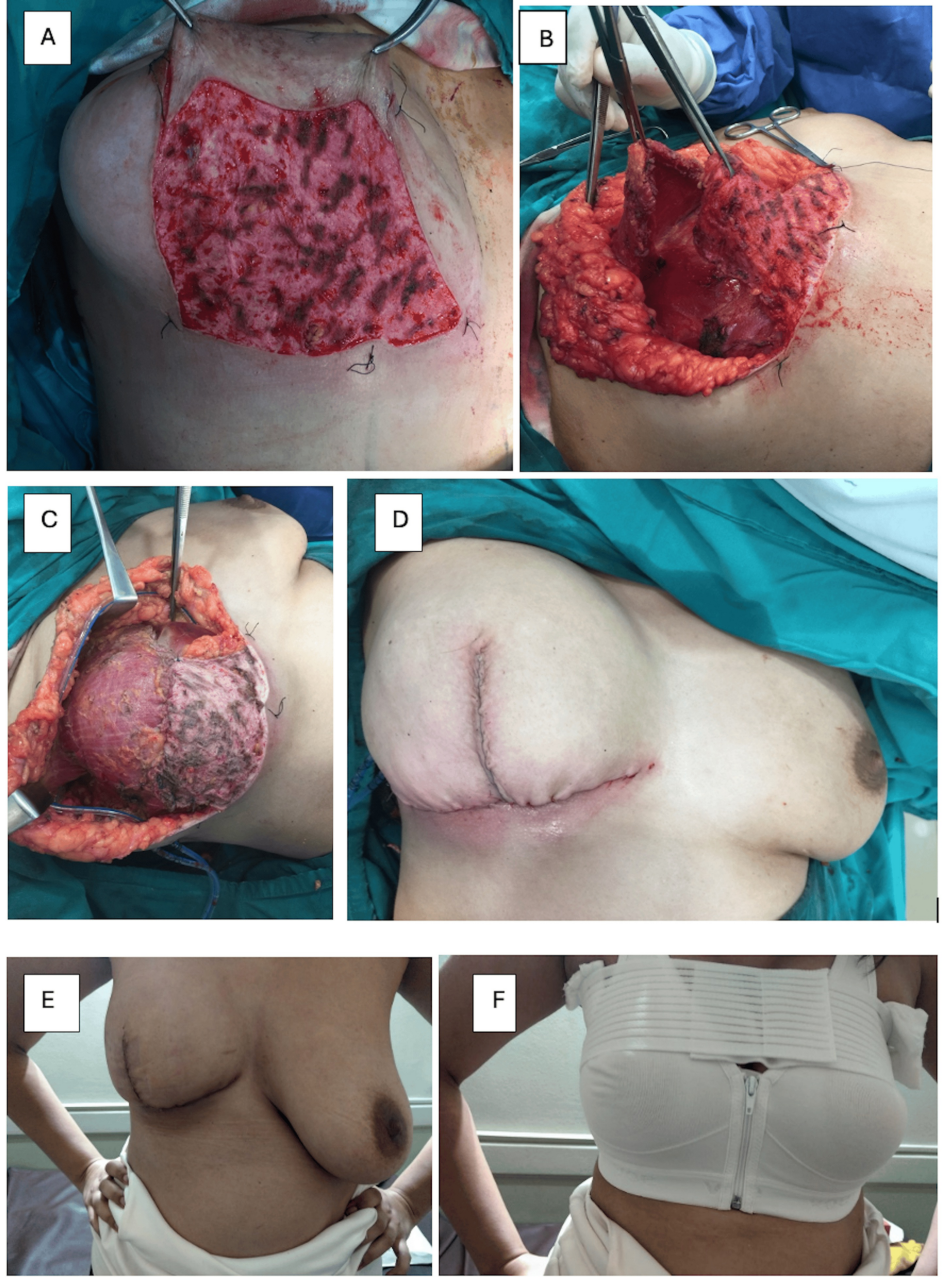
Good cosmetic results of SRM and subpectoral implant reconstruction: (A) De-epithelization of the dermal sling; (B) Preparing the dermal sling and detaching the pectoralis major muscle; (C) Subpectoral insertion of the implant and suturing the muscle to the dermal sling; (D) Intraoperative picture after closure; (E) One month postoperative; (F) Final picture with supportive bra. SRM: skin-reducing mastectomy

Assessment of aesthetic outcomes was carried out by clinical breast examination to assess the breast shape and contour, definition of the IMF, breast consistency, and overall results. Regarding breast shape and contour, 13 cases demonstrated excellent results (65%), five cases exhibited good results (25%), one case had a fair result (5%), and one case had a poor result (5%). The IMF was well-defined in 19 cases (95%), while only one case had an ill-defined IMF (5%). The breast consistency was soft in all the studied cases (100%). None of the studied cases had a firm or hard breast consistency. The overall results were excellent in 12 cases (60%), good in five cases (25%), and fair in two cases (10%), while it was poor in only one case (5%) (Table 3, Figures 4-6).

**Table 3:** Distribution of the studied cases according to overall result (N = 20)

Overall result	Frequency	Percentage
Excellent	12	60%
Good	5	25%
Fair	2	10%
Poor	1	5%

Regarding patient satisfaction, 12 patients (60%) expressed extreme satisfaction, six (30%) were satisfied, and two (10%) were less satisfied. None of the studied cases were dissatisfied (Table 4). Table 5 shows that the prepectoral group had better overall results, with seven patients rated as excellent compared to five in the subpectoral group. However, as seen in Table 6, this didn’t translate into a significant difference in patient satisfaction. Both groups had similar levels of satisfaction, with 60% of patients in each group feeling extremely satisfied. 

**Table 4:** Distribution of the studied cases according to patient satisfaction (N = 20)

Patient satisfaction	Frequency	Percentage
Extremely satisfied	12	60%
Satisfied	6	30%
Less satisfied	2	10%
Dissatisfied	0	0%

**Table 5:** Relation between positioning of implant and overall result (N = 20) X^2^: Chi square test; MC: Monte Carlo test; p: p value for comparing between Subpectoral and Prepectoral *: Statistically significant at p ≤ 0.05

Overall result	Positioning of implant						X^2^	^MC^p
	Subpectoral (n = 10)			Prepectoral (n = 10)				
	Frequency		Percentage	Frequency		Percentage		
Excellent	5	50%		7	70%		7.758^*^	0.022^*^
Good	5	50%		0	0%			
Fair	0	0%		2	20%			
Poor	0	0%		1	10%			

**Table 6:** Relation between positioning of implant and patient satisfaction (N = 20) X^2^: Chi square test; MC: Monte Carlo test; p: p value for comparing between Subpectoral and Prepectoral

Patient Satisfaction	Positioning of implant						X^2^	^MC^p
	Subpectoral (n = 10)			Prepectoral (n = 10)				
	Frequency		Percentage	Frequency		Percentage		
Extremely satisfied	6	60%		6	60%		2.303	0.374
Satisfied	4	40%		2	20%			
Less satisfied	0	0%		2	20%			
Dissatisfied	0	0%		0	0%			

## Discussion

In 1991, SSM was introduced by Toth and Lappert as an attempt for skin preservation to facilitate immediate breast reconstruction [5]. However, it is technically challenging to perform SSM and primary reconstruction in large and ptotic breasts [6]. In the early 1990s, Bostwick introduced a modified technique of skin-sparing mastectomy to remove the breast via an inverted T or Wise-pattern incision [16]. This technique involved making a de-epithelized dermal flap and suturing it to the lower fibres of the pectoralis major muscle to create a pouch for covering the implant. It was used during prophylactic mastectomy. In 2002, Skoll and Hudson [17] and Hammond et al. [18] used this approach for breast reconstruction in breast cancer patients. Hammond et al. used the Wise-pattern technique together with the insertion of a temporary tissue expander under the dermo-muscular pouch with subsequent placement of the implant in a delayed stage [18]. Then, in 2006, this technique was modified by Nava et al., who popularized the term ‘‘skin-reducing mastectomy’’ and used this technique with immediate single-stage breast reconstruction using an implant to be inserted under the dermo-muscular pouch [8].

The current study included 20 female patients who underwent SRM via Wise-pattern skin incision and immediate implant-based breast reconstruction. All the patients had breast cup sizes D and above. The mean implant size was 477.25 cc, ranging from 325 cc to 615 cc. The implant was inserted above the pectoralis major covered solely by the dermal sling in 10 cases and partially subpectoral by suturing the dermal sling to the lower pectoralis major fibres in the remaining 10 cases. Nava et al. conducted a study in Milan that involved 28 patients between 2001 and 2004 [8]. The mean size of implant used was 433 cc ranging from 195 cc to 620 cc. All the implants were inserted submuscularly by dissecting along the lateral border of the pectoralis major muscle and dividing its inferior border to suture it to the dermal sling. Irwin et al. conducted a retrospective study on 104 patients who underwent mastectomy and reconstruction between October 2008 and October 2012 [19]. They concluded that SRM and one-stage DTI reconstruction was safe and should be considered in selected patients with enough size of skin envelope.

In our study, we did not use biological or synthetic meshes in any of our cases. De Vita et al. conducted a retrospective study of 88 cases [20]. They used a musculo-dermal pouch to cover the implant under the pectoralis major muscle in all cases. In addition, they used ADM in 14 cases to increase the width of the pouch when the dermal flap was not adequate to cover the implant. They concluded from the study that SRM with immediate placement of an implant is an indispensable choice for the management of women with breast cancer with large and ptotic breasts.

On the other hand, the SRM technique was not preferred by some surgeons. Schneider et al. conducted a study on 85 patients who underwent nipple sparing mastectomy (NSM) [21]. They analyzed a subgroup of patients with large ptotic breasts, defined as cup size C or greater, distance from the sternal notch to the nipple more than 24 cm, and grade 2 or 3 breast ptosis. Of the 85 patients, 19 fit the inclusion criteria. There was only one case of nipple necrosis in a patient who had previously received radiotherapy (5%), one case suffered from postoperative haematoma (5%), and none of the cases was complicated by flap necrosis. They concluded from the study that the rate of areolar necrosis in NSM in large ptotic breasts was only 5%, which is similar to that of other studies. Thus, it is not necessary to reduce the skin envelope in such patients.

In the current study, nipple- areola preservation was done in five patients. Four cases had primary NAC reconstruction during the mastectomy operations. They had an intra-operative frozen section of the retro-areolar disc. Nipple-areola graft was done for three cases with a resultant excellent cosmetic outcome. The fourth patient underwent preservation of the NAC based on superior and inferior dermal pedicles; however, the areola became necrotic one week after the operation and had to be removed in a second session. One patient had delayed nipple reconstruction in a second session using local skin flaps and also displayed an excellent aesthetic result. The most frequent complication encountered in our study was delayed healing of the T-junction, encountered in two patients and managed conservatively. One patient had implant displacement noticed in the first follow-up visit and needed surgical management in a second session by inserting the implant under the pectoralis major and suturing it to the dermal sling. None of the studied cases suffered from infection, hematoma, or capsular contracture. Thus, there were no cases of implant loss.

Nava et al. performed single-stage nipple reconstruction for three patients after intra-operative frozen section [8]. Local skin flaps were used for one case, and the other two cases had nipple-areola skin grafts; however, one of the cases, who was a heavy smoker, suffered from graft failure. Six patients underwent nipple reconstruction in a second stage six months after the first operation. They reported in his study 20% complications. Superficial epidermolysis or wound dehiscence was encountered in six patients and was initially managed conservatively by frequent wound dressings. Two cases responded to the conservative management; however, implant removal was needed in the remaining four cases.

De Vita et al. performed immediate nipple graft for 82 breasts from the whole studied 88 cases [20]. The remaining six patients showed positive retro-areolar disc on frozen section; thus, they were not grafted. He reported total loss of the NAC in five patients and partial loss in five patients, which healed by second intention. Other complications encountered in his study included four cases of superficial epidermolysis, three cases of wound dehiscence, and two cases of implant extrusion that needed the removal of the implants. In addition, 15 patients suffered from capsular contracture and required an exchange of the implants.

This study has several limitations that should be acknowledged. First, the sample size was relatively small (20 patients), which may limit the generalizability of the findings. Additionally, the median follow-up of 30 months may not be sufficient to fully assess long-term complications such as capsular contracture, implant failure, aesthetic changes over time, or the potential long-term outcomes of NAC grafting, including pigmentation changes or sensory loss. Furthermore, the study was conducted during the COVID-19 pandemic, which impacted patient recruitment and may have influenced the findings. Notably, the study was conducted on a group of patients who were not indicated for radiotherapy, which may have contributed to the low complication rate observed. Further research is needed to evaluate the impact of radiotherapy on surgical outcomes and complication rates. Nevertheless, this research provides valuable insights into a technique that can be safely implemented for breast reconstruction, and the results highlight its potential for achieving satisfactory cosmetic outcomes without the need for additional costly materials like ADM or synthetic meshes, especially in low-income countries with limited resources.

## Conclusions

Based on our findings, SRM and DTI breast reconstruction is a feasible technique that can be safely used in patients with large and ptotic breasts as it allows for the reduction of the skin envelope. Thus, it preserves the vascularity of the mastectomy skin flaps and achieves a satisfactory cosmetic result in patients with macromastia. In addition, the inferior dermal sling can be used to cover the implants; thus, it allows for a single-stage implant-based breast reconstruction without the need for ADM or synthetic meshes. Nipple-areola graft is a safe technique for NAC preservation with SRM in patients with sizable skin envelope after confirmation that the retro-areolar disc is negative by frozen section.
